# Prostaglandin F2α synthase in *Trypanosoma cruzi* plays critical roles in oxidative stress and susceptibility to benznidazole

**DOI:** 10.1098/rsos.170773

**Published:** 2017-09-20

**Authors:** Paola García-Huertas, Ana María Mejía-Jaramillo, Carlos Renato Machado, Anna Cláudia Guimarães, Omar Triana-Chávez

**Affiliations:** 1 Grupo Biología y Control de Enfermedades Infecciosas-BCEI, Instituto de Biología, Universidad de Antioquia, Calle 70 52-21, Medellín, Colombia; 2 Departamento de Bioquímica e Inmunologia, Universidade Federal de Minas Gerais, Belo Horizonte, Minas Gerais, Brazil

**Keywords:** benznidazole resistance, mechanism of action, *Trypanosoma cruzi*, functional genomics, prostaglandin F2α synthase

## Abstract

Nifurtimox (Nfx) and benznidazole (Bz) are the current drugs used for the treatment of Chagas disease. The mechanisms of action and resistance to these drugs in this parasite are poorly known. Prostaglandin F2α synthase or old yellow enzyme (OYE), an NAD(P)H flavin oxidoreductase, has been involved in the activation pathway of other trypanocidal drugs such as Nfx; however, its role in the mechanism of action of Bz is uncertain. In this paper, we performed some experiments of functional genomics in the parasite *Trypanosoma cruzi* with the aim to test the role of this gene in the resistance to Bz. For this, we overexpressed this gene in sensitive parasites and evaluated the resistance level to the drug and other chemical compounds such as hydrogen peroxide, methyl methanesulfonate and gamma radiation. Interestingly, parasites overexpressing OYE showed alteration of enzymes associated with oxidative stress protection such as superoxide dismutase A and trypanothione reductase. Furthermore, transfected parasites were more sensitive to drugs, genetic damage and oxidative stress. Additionally, transfected parasites were less infective than wild-type parasites and they showed higher alteration in mitochondrial membrane potential and cell cycle after treatment with Bz. These results supply essential information to help further the understanding of the mechanism of action of Bz in *T. cruzi*.

## 1. Background

Nifurtimox (Nfx) and benznidazole (Bz) are the current drugs used for the treatment of Chagas disease. Their use is characterized by toxicity and low efficacy in the chronic stage of the disease, and cases refractory to the treatment are commonly reported; some of them are associated with the drug resistance of its causative agent, the protozoa *Trypanosoma cruzi* [1].

The mode of action of nitroheterocyclic compounds appears to involve the metabolic activation of the compounds initiated through the reduction of the drugs' nitro groups mediated by a mitochondrial NADH-dependent type-I nitroreductase (NTR I), which uses FMN as a cofactor [2]. NTR I metabolizes Bz and reduces it to a toxic compound called glyoxal, which forms adducts with guanosine and prevents the formation of new DNA strands [2]. Several studies have shown mutagenic and genotoxic effects of Bz in different cell models, double-stranded breaks in treated parasites possibly caused by nucleotide oxidation [3] and cell cycle arrest mainly in G0/G1 [4,5].

On the other hand, it has been proposed that the mechanism of action of Bz is related to the induction of oxidative stress, caused by reactive oxygen species (ROS) [6]. In comparison with mammalian cells, the parasite's defence mechanisms against oxidative stress are defective [7]. No catalase or glutathione peroxidase activity has been detected in *T. cruzi*, and superoxide dismutase activity is very much diminished [8,9]. Therefore, *T. cruzi*'s main defence mechanism against free radicals is trypanothione, characteristic of all trypanosomatids. This hypothesis is supported by recent studies where Bz induces 7,8-dihydro-8-oxoguanine (8-oxoG) formation in *T. cruzi*, which is the most common and best-characterized lesion created by ROS that in large proportion affects the DNA and induces the death of the parasite [3,10]. Other evidence is the resistance to hydrogen peroxide (H_2_O_2_), where it has been shown that H_2_O_2_-resistant parasites are also resistant to Bz [3,10] indicating a close relationship between tolerance to oxidative stress and the effect of Bz. Despite these discoveries, the mechanism of action of this drug is far from being understood. To date, only NTR I has been verified to participate in the activation of Bz by functional genomics, through overexpression in resistant parasites and knockdown in sensitive parasites [11].

Prostaglandin F2α synthase or old yellow enzyme (OYE), an other NAD(P)H flavin oxidoreductase, similar to NTR I, has been involved in the activation pathway of other trypanocidal drugs such as Nfx but not Bz [12]. Different studies have shown that OYE was found to be downregulated in resistant parasites, analysing RNA and protein [13,14]. Accordingly, we found recently that this gene is downregulated in parasites with natural resistance to Bz [15]. However, Mejía-Jaramillo *et al*. [16], found that Gal61R parasites with induced Bz resistance, which lack NTR I enzyme, had OYE upregulated. In order to analyse the real role of OYE in *T. cruzi* Bz resistance, in this study we overexpressed OYE in sensitive and resistant parasites and evaluated their response to Bz. Taking into account that Bz induces oxidative stress and genetic damage in the parasites, we also performed some experiments directed to understanding the response of parasites overexpressing OYE to H_2_O_2_ and compounds causing DNA damage. This study, together with other genomic research, provides a good approach to understanding the resistance to Bz and the mode of action of this drug.

## 2. Methods

### 2.1. Parasites

The *Trypanosoma cruzi* TcI 61Scl11 clone susceptible to Bz, with an inhibitory concentration 50 (IC_50_) of 11.7 µM, and the TcI 61Rcl4 clone resistant to Bz, with IC_50_ of 47.3 µM, were used in this study [11,16]. Epimastigotes were cultivated in supplemented Roswell Park Memorial Institute (RPMI) 1640 medium at 28°C [17], and the cultures were maintained in exponential growth by passaging every 7 days [18]. Transfected *T. cruzi* parasites were maintained with 100 µg ml^−1^ of G418.

### 2.2. Cloning of the OYE gene and transfection

The ORF of *OYE* (XM_816510.1) was amplified using DNA from *T. cruzi* with specific primers (F-5′ATGGCGACGTTCCCTGAACTTCTG 3′ and R-5′ TTATTTGTTGTACGTCGGGTAATCG 3′). Fragments were digested and ligated into the vector pTEX [19], and the resulting construct was used to transform *Escherichia coli* DH5α. The construct was purified and confirmed by sequencing. For electroporation, 6 × 10^7^ parasites ml^−1^ in the logarithmic growth phase were suspended in electroporation buffer (132 mM NaCl, 8 mM KCl, 8 mM Na_2_HPO_4_, 1.5 mM KH_2_PO_4_, 0.5 mM MgOAc_2_, 90 µM CaOAc_2_, adjusted to pH 7.0 with acetic acid, filter sterilized) and transfected with 50 µg of plasmid in a BioRad GenePulse II electroporator with two electric pulses of 1.5 kV, 25 µF, 50 ohms, in a 2 mm gap cuvette. The selection of transfectants was performed with G418 (100 mg ml^−1^). Transfected parasites with the construct pTEX-GFP were used as a control. Overexpression was verified by qRT-PCR [16], Northern blot analysis was performed according to the conditions previously described by Kim *et al*. [20], with minor adjustments, and western blot as is described below.

### 2.3. Susceptibility to Bz, H_2_O_2_, MMS and gamma irradiation

Transfected epimastigotes overexpressing OYE and GFP (control) were maintained in the logarithmic phase (5 × 10^6^ cells ml^−1^) during 7 days. A total of 1 × 10^7^ cells ml^−1^ were used to monitor the daily growth of the parasites for 6–7 days and to perform all the survival assays. For testing the sensitivity to Bz, parasite cultures were treated with 0 and 240 µM Bz, and cells were counted after 72 h, following the recommendations previously described [3]. For testing the sensitivity to H_2_O_2_, the parasites were treated with 0 and 150 µM of H_2_O_2_ for 20 min, and the cells were then centrifuged and suspended in fresh medium and counted after 72 h [10]. To evaluate the effect of DNA alkylating agent, the parasites were treated with 0 and 1.5 mM of methyl methanesulfonate (MMS) for 1 h, and the cells were then centrifuged and suspended in fresh medium and counted daily for 6 days. The effect of gamma irradiation was evaluated after the cells were exposed to a dose of 500 Gy (1578 Gy h^−1^ for 20 min) in a cobalt (^60^Co) irradiator located at Centro de Desenvolvimento da Tecnologia Nuclear (CDTN), Belo Horizonte, Brazil, and were then incubated and counted daily for 13 days. For all experiments, the cell numbers were determined in a cytometry chamber using the erythrosine vital stain (phosphate-buffered saline (PBS) 1× + erythrosine 0.4%) to differentiate living and dead cells. Experiments were performed in triplicate. Data were tested for normality and differences between the treated and non-treated parasites were examined using analysis of variance (ANOVA) in the GraphPad Prism v. 5.0a software.

### 2.4. Mitochondrial membrane potential analyses

A total of 1 × 10^7^ epimastigotes ml^−1^ were treated or not with 120 µM Bz and incubated for 48 h at 28°C. For cell cycle analysis, the cells were washed with PBS and fixed with ice-cold 70% ethanol at 4°C for 24 h. Subsequently, the cells were washed once with PBS and suspended in PBS containing 10 µg ml^−1^ propidium iodide and 10 µg ml^−1^ RNAse A. After 45 min of incubation at 37°C, the cells were analysed by flow cytometry. For mitochondrial membrane potential measurements, the parasites were washed with PBS and suspended in PBS containing 0.8 nM 3,3′-dihexyloxacarbocyanine iodide (DiOC6). The suspension was incubated in the dark at room temperature for 15 min before flow cytometric analysis.

Analysis for both experiments was performed using the BD FACS Calibur equipment, and the data were analysed by Flowjo v. X.0.7 software. The cell cycle data were analysed by comparing the cell cycles of treated and non-treated parasites. Mitochondrial membrane potentials were analysed by comparing the mean fluorescence intensity of treated and non-treated parasites. Data were tested for normality, and differences in the treated and non-treated parasites were examined using paired *t*-test in the GraphPad Prism v. 5.0a software.

### 2.5. Infection assay

To generate trypomastigotes, Vero cells cultured in DMEM/10% fetal bovine serum at 37°C in 5% CO_2_ were infected with epimastigotes in the stationary phase, at which point they differentiated into metacyclic trypomastigotes. Trypomastigotes emerged between day 7 and 10, and this homogeneous population was used in the quantitative infection experiments. A total of 30 000 cells were infected at a ratio of 3 trypomastigotes per mammalian cell. Following incubation at 37°C for 24 h, extracellular parasites were removed by several washes. After 48 h, the cells were stained with Giemsa and 500 cells by treatment were counted. The number of amastigotes and the percentage of infection was obtained from five experiments repeated in triplicate. The differences between the control parasites (pTEX-GFP) and pTEX-OYE parasites were examined using paired Dunnett's test in the GraphPad Prism v. 5.0a software.

### 2.6. Western blotting analysis

The polyclonal antibodies anti-SOD-A (superoxide dismutase A), anti-TR (trypanothione reductase) and anti-OYE were obtained as previously described [21–23]. Epimastigotes (1.0–2.0^7^ cells ml^−1^) were incubated or not (control) with Bz (120 µM) at 28°C for 48 h. The cells were harvested by centrifugation (3000 rpm, 10 min) and suspended in 80 µl of PBS/1 mM MgCl_2_, and an equal volume of lysis buffer (50 mM Tris HCl pH 7.4, 1% Tween 20, 150 mM NaCl, 1 mM EGTA, 1 mM Na_3_VO_4_, 1 mM NaF, 0.1 mM PMSF, aprotinin 1 mg ml^−1^, leupeptin 1 mg ml^−1^) was added. The suspension was sonicated (Bandelin Sonoplus Homogenisatoren) for 10 cycles of 1 s, with an interval of 1 s and 30% max amperage. The material was kept for 2 h on ice and subsequently centrifuged (13 000*g*, 4°C, 15 min). An equal volume of loading buffer was added to the protein extract (100 mM Tris-HCl, pH 6.8, 4% SDS, 0.02% bromophenol blue, 20% glycerol, 200 mM beta-mercaptoethanol), and the samples were heated at 96°C for 4 min [24]. The protein concentration was determined by the Bradford technique in samples without loading buffer. The protein extracts (30 µg) were separated and electroblotted onto a nitrocellulose membrane using the Trans-Blot SD Semi-Dry Electrophoretic Transfer Cell (BioRad, CA, USA). The membranes were blocked by incubation with 5% instant nonfat dried milk in PBS plus 0.05% Tween 20 (PBS-T) for 1 h and washed and incubated in the presence of polyclonal antibodies raised against anti-SOD-A, anti-TR or anti-OYE, according to the experiment, for 2 h. After three washes of 10 min with PBS-T, the membranes were incubated with HRP-linked anti-rabbit IgG (Cell Signaling Technology, MA, USA, 1 : 5000 dilution) for 1 h at room temperature and washed three times with PBS [25,26]. The bands were visualized using the Super Signal Detection Kit (Thermo Scientific, Pierce, IL, USA). The data were analysed using the ImageJ program and normalized using a protein band detected with Ponceau staining and glyceraldehyde 3-phosphate dehydrogenase (GAPDH) as loading control for SOD and TR, and OYE, respectively. Statistical analyses were performed with GraphPad Prism v. 5.0a software. Three independent experiments were performed.

## 3. Results

### 3.1. Parasites overexpressing OYE are more sensitive to Bz and oxidative stress

**Figure 1.**
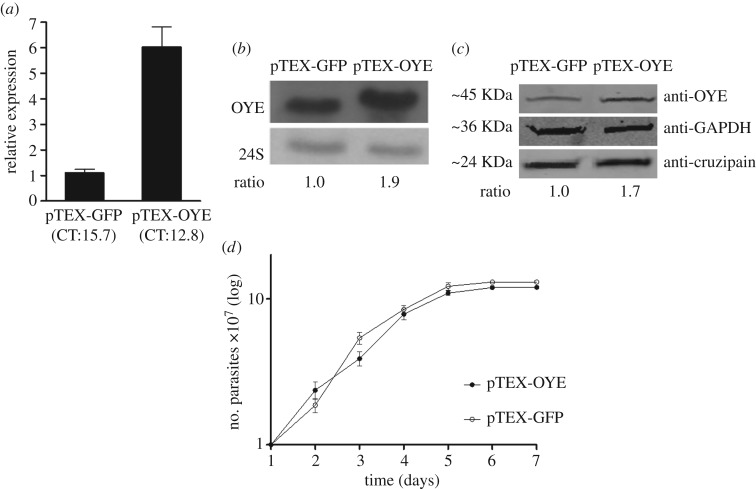
Overexpression of OYE in sensitive parasites and growth curve. (*a*) Quantification by qRT-PCR of mRNA levels among pTEX-OYE and pTEX-GFP parasites. Data were normalized with the expression of the reference gene (ribosomal 24S). CT, threshold cycle; error bars represent standard deviations. (*b*) OYE overexpression analysis in sensitive parasites by northern blot. The ribosomal 24S gene was used as the normalizer. (*c*) OYE protein overexpression analysis in pTEX-OYE parasites by western blot. GAPDH and cruzipain proteins were used as load controls. The quantification was performed with GAPDH as the normalizer. (*d*) Growth curve of transfected parasites with OYE (pTEX-OYE) and control parasites (pTEX-GFP) during 7 days. Error bars represent standard deviations from three independent experiments.

Mejia-Jaramillo *et al*. [16] reported that Bz resistant 61R cl4 parasites had OYE upregulated. First, we wanted to evaluate if OYE enzyme was overexpressed in these parasites. In fact, we found that OYE was expressed around twice as much in resistant parasites (electronic supplementary material, figure S1). With this in mind, we overexpressed OYE gene in susceptible and resistant *T. cruzi* parasites with the aim to evaluate its role in Bz resistance. The overexpression in susceptible parasites to Bz was confirmed at RNA level using qRT-PCR (figure 1*a*) and Northern blot (figure 1*b*); and by western blot (figure 1*c*). pTEX-OYE parasites presented 1.7 times higher levels of OYE than control parasites, pTEX-GFP. The growth curve showed no statistically significant differences in the growth kinetics between both parasites (figure 1*d*). However, it was found that pTEX-GFP parasites required 21.5 h to double growth, whereas pTEX-OYE parasites required 24.5 h. Interestingly, pTEX-GFP parasites did not show statistically significant differences compared with wild-type parasites, and for this reason we decided to use these parasites as control in all experiments. Intriguingly, the 61R cl4 resistant parasites overexpressing this gene were not viable after three months of follow-up.

**Figure 2.**
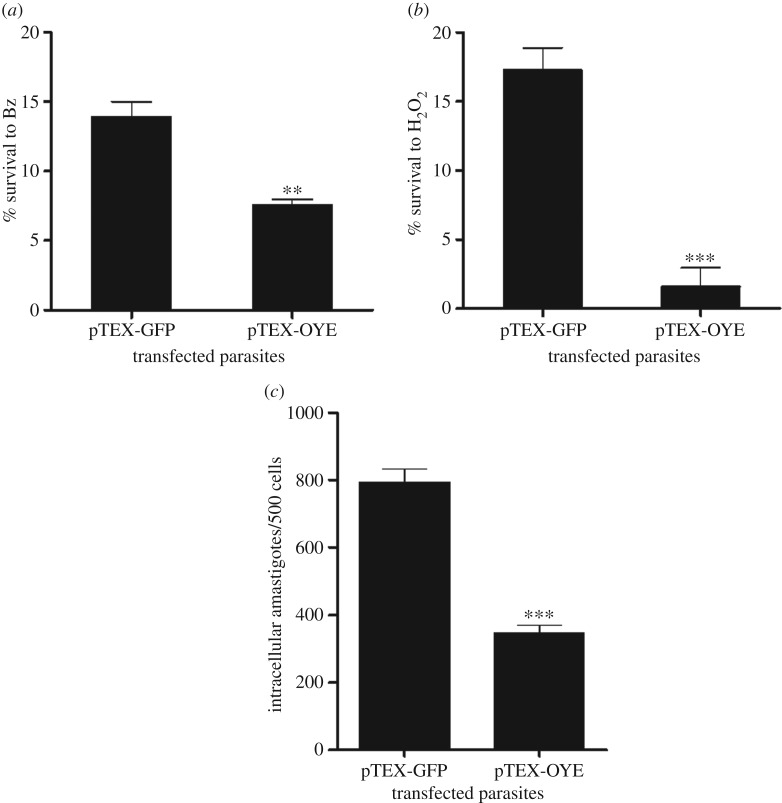
Biological effects of OYE overexpression in resistance to Bz and H_2_O_2_, and cell infection in *T. cruzi*. Survival of pTEX-OYE and pTEX-GFP epimastigotes after treatment with 120 µM Bz (*a*) and 100 µM H_2_O_2_ (*b*). (*c*) Vero cell infection with pTEX-GFP and pTEX-OYE trypomastigotes. Bars represent intracellular amastigotes obtained from five independent experiments in triplicate. Asterisks indicate statistical difference between pTEX-GFP and pTEX-OYE, *** *p* < 0.001, ** *p* < 0.01; error bars represent standard deviations.

To determine the response to Bz and peroxide, we evaluated a concentration range of 0 µM to 240 µM for Bz and 0 µM to 150 µM for H_2_O_2_. The survival assay showed that pTEX-OYE parasites were approximately 2 times more susceptible to Bz and 10 times more susceptible to H_2_O_2_ in comparison with pTEX-GFP parasites. Figure 2*a*,*b* shows the response to 120 µM Bz and 100 µM H_2_O_2_, respectively.

### 3.2. Infection of Vero cells is affected in transfected parasites

Given that transfected parasites were more sensitive to Bz and H_2_O_2_, we decided to evaluate the infectivity of these parasites in Vero cells because the infection is related to oxidative stress. No statistically significant differences between infection rates of pTEX-OYE and pTEX-GFP parasites were observed, although the number of intracellular parasites was significantly lower for the pTEX-OYE parasites (figure 2*c*).

### 3.3. Antioxidant enzymes are changed in response to OYE overexpression

**Figure 3.**
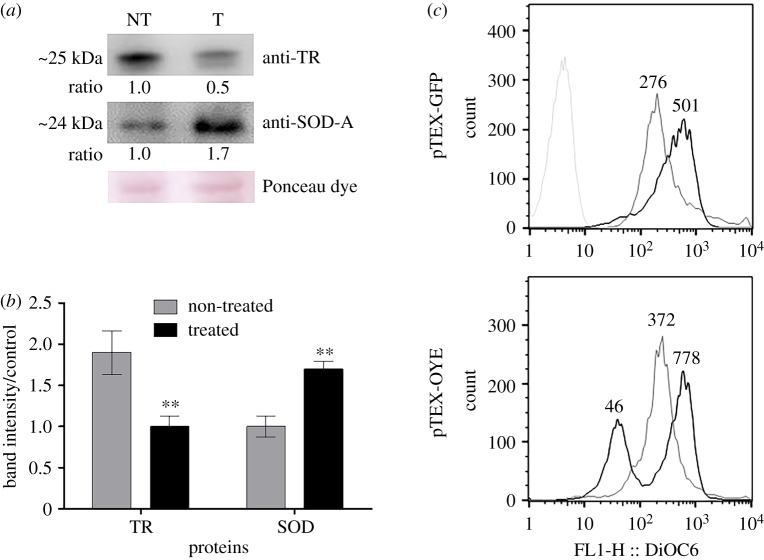
Alteration of oxidative stress in parasites overexpressing OYE after treatment with Bz. (*a*) Western blot analysis of treated (T) and non-treated (NT) pTEX-OYE parasites with 120 µM Bz using anti-TR and anti-SOD-A. Load control was verified by Ponceau staining. (*b*) The bars represent the band intensity of proteins TR and SODA in treated and non-treated pTEX-OYE parasites. (*c*) Values represent the mean fluorescence intensity of the dye DiOC6 within mitochondria of treated and non-treated parasites with 120 µM Bz obtained by flow cytometry. Parasites treated with Bz are shown in black, non-treated parasites in grey and parasites without dye in light grey. Asterisks indicate statistical difference between pTEX-GFP and pTEX-OYE, ** *p* < 0.01; error bars represent standard deviations.

Because the sensitivity to oxidative stress was affected in the transfected parasites, we tested if the levels of antioxidant enzymes changed in the parasites after the oxidative stress generated by treatment with 120 µM Bz. Band densitometry of the western blot assay showed that the Bz treatment decreased the TR levels by approximately 2 times in the pTEX-OYE parasites. Conversely, the SOD-A levels were increased approximately 1.7 times in these parasites after the same treatment (figure 3*a*,*b*).

### 3.4. Mitochondrial membrane potential is altered in parasites overexpressing OYE after treatment with Bz

To determine if the sensitivity to oxidative stress caused by Bz is due to alterations in mitochondrial function, an analysis of the mitochondrial membrane potential in the Bz-treated and non-treated parasites was performed (figure 3*c*). The pTEX-GFP parasites treated with Bz had a hyperpolarization of the mitochondrial membrane potential, represented by a mean fluorescence intensity of 501 compared to 276 obtained in non-treated parasites. In contrast, pTEX-OYE parasites treated with Bz showed two distinct populations, one with depolarized mitochondrial membrane potential (mean fluorescent intensity of 46.1) and the other with a hyperpolarized membrane potential (mean fluorescent intensity of 778), showing statistically significant differences when compared with the non-treated parasites (mean fluorescent intensity of 372). These results showed that the mitochondrial function of the pTEX-OYE parasites was altered after treatment with Bz.

### 3.5. Parasites overexpressing OYE have increased sensitivity to genetic damage

**Figure 4.**
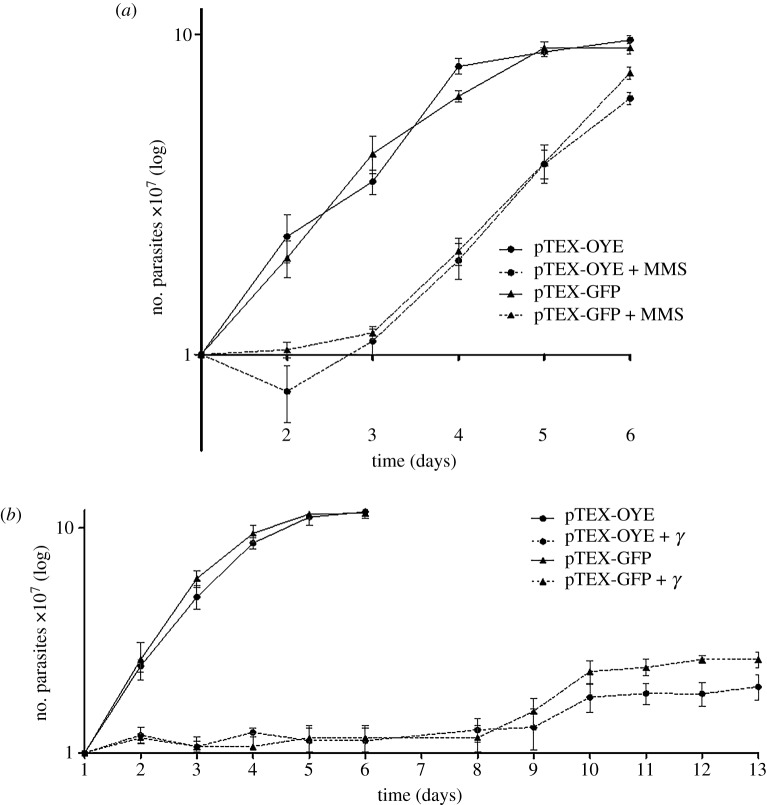
Effect of OYE overexpression in response to genetic damage caused by MMS and gamma radiation. (*a*) Represents the number of parasites obtained after treatment with 1.5 mM MMS. The solid and dotted lines correspond to MMS non-treated and treated parasites, respectively. (*b*) Represents the growth of parasites after exposure to 500 Gy gamma radiation. The solid lines correspond to non-irradiated parasites, and dotted lines correspond to irradiated parasites. Error bars represent the standard deviations of three independent experiments.

In order to evaluate the response of pTEX-OYE parasites to agents causing genetic damage, we evaluated the growth of these parasites in the presence of MMS and gamma radiation (figure 4). The non-treated controls grew similarly and reached the stationary phase after day 5, as was expected. The growth of transfected and non-transfected parasites was affected during the first two days post-treatment with MMS, although significant decrease in the number of pTEX-OYE parasites in comparison to pTEX-GFP parasites after day 6 post-treatment was observed (figure 4*a*). In the same way, the gamma radiation promoted a growth arrest for approximately 8 days with a slight recovery at day 9 post-treatment for both groups of parasites, but in pTEX-OYE transfected parasites the recovery was smaller (figure 4*b*).

### 3.6. Bz affects the cell cycle in pTEX-OYE treated parasites

**Figure 5.**
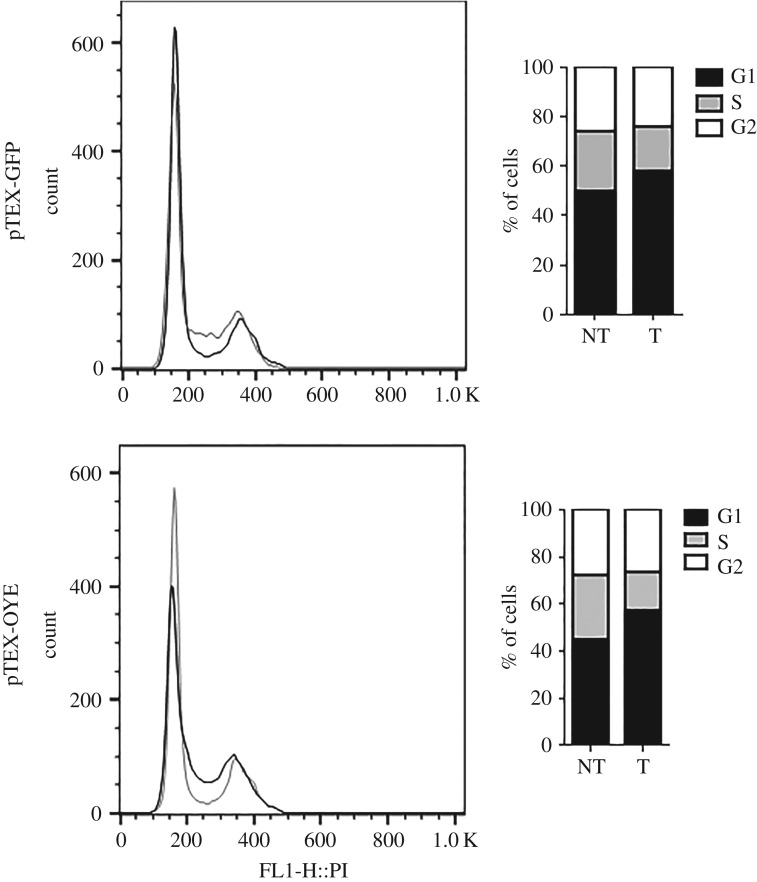
Alteration of cell cycle of transfected parasites after treatment with Bz. The cell cycle profile of parasites before and after treatment with 120 µM Bz for pTEX-GFP and pTEX-OYE parasites. The black and grey lines represent the cell cycle profile of non-treated and treated parasites, respectively. Bars on the right represent the percentage of Bz-treated (T) and non-Bz treated (NT) cells in each cell cycle phase (G1, S, G2/M).

Considering that pTEX-OYE parasites were more sensitive to Bz and genetic damage, the effect on the cell cycle of these parasites was assessed after treatment with 120 µM Bz for 48 h. The pTEX-GFP parasites treated with Bz showed a small accumulation of cells in the G1 phase, with approximately 8% more cells than non-treated parasites, and a decrease of cells that were mainly in S phase, where a difference of 5% was found with non-treated parasites. Likewise, pTEX-OYE parasites accumulated a higher percentage of cells in G1 phase after treatment, as well as a reduction of cells in S phase (figure 5).

## 4. Discussion

The scarce knowledge about the mechanisms of action of Nfx and Bz is an obstacle to the progress of controlling the resistance of *T. cruzi* and the identification of genes involved in this process, as well as the search for new therapeutic options for treating Chagas disease. Thus, elucidating the mechanisms of action of these drugs would give understanding of the different metabolic pathways involved in the acquisition of the resistance of *T. cruzi* and help to find other drugs that are effective against the parasite or enhance the effect of Bz by combined therapy.

In this paper, we performed a functional genomic analysis of prostaglandin F2 alpha synthase (OYE) to identify the real role of this protein in the mode of action of Bz. This enzyme, an oxidoreductase, has been the subject of various studies which have shown differences in regulation between sensitive and resistant parasites to this drug. Murta *et al*. [14] found that this protein was downregulated in resistant parasites due to the deletion of three copies of the gene [14]. Likewise, by proteome analysis, it was found that OYE was under-expressed in resistant parasites [13]. Conversely, Mejia *et al*. [16] showed that the OYE gene was upregulated in resistant parasites [16]. Interestingly, this was attributed to the absence of functional NTR I in these parasites [11]. Thus, it has been proposed that OYE also participates in the activation of Bz generating toxic radical anions in the parasite [12]. It is important to highlight that the resistant parasites obtained by Murta *et al*. [14] were exposed to 220 µM Bz, and they lost three copies of the OYE gene, whereas the resistant parasites obtained by Mejia *et al.* [16] were exposed only to 50 µM, and lost one copy of NTR I and overexpressed OYE [14,16]. This suggests that when *T. cruzi* parasites are exposed to Bz, first they inactivate NTR I and subsequently, at higher drug concentration, they lose OYE, as was demonstrated by Murta *et al*. [14]. In this sense, our results support the role of OYE in activating Bz. We found that parasites overexpressing OYE were more sensitive to Bz and H_2_O_2_. Interestingly, when we tried to overexpress OYE in resistant parasites, which already have OYE overexpressed by about 1.9 times (electronic supplementary material, figure S1), they did not survive, probably because they are not able to deal with the oxidative stress level and genetic damage occasioned by the overexpression of this protein. On the other hand, we also found that parasites overexpressing OYE were less infective than GFP-transfected parasites, evidenced by a smaller number of intracellular amastigotes. These results are not surprising due to previous results showing that the *T. cruzi* parasites resistant to oxidative stress, caused mainly by H_2_O_2_, were more infective than sensitive parasites [27].

In addition, western blot analysis showed that the parasites overexpressing OYE have decreased TR and increased SOD after treatment with Bz. A previous study showed that overexpression of SOD in *T. cruzi* increased the sensitivity to the trypanocidal agents Bz and gentian violet, due to the accumulation of hydrogen peroxide [28]. This result coupled with decreased TR, which protects the parasite from oxidative stress, may reflect an imbalance in the antioxidant defence of the transfected parasites.

Considering that Bz metabolism appears to be conducted in the mitochondria and during this process free radicals that induce oxidative stress affect the function of this organelle, the mitochondrial membrane potential was assessed after Bz treatment. Supporting our previous results related to oxidative stress caused by Bz, an alteration of the mitochondrial membrane potential after treatment with Bz was observed. This alteration of the mitochondrial membrane potential has been associated with an early event of programmed cell death mainly induced by toxic oxygen intermediates [29]. Although there are few studies that relate the alteration of the mitochondrial function to the effect of Bz, it is possible to establish that the alteration of mitochondrial membrane potential in parasites overexpressing OYE may result from the reactive oxygen intermediates generated by the metabolism of Bz.

On the other hand, it has been proposed that Bz induces genetic damage in *T. cruzi* mainly associated with double-strand breaks [3,30]. To address this hypothesis, the response to MMS, an alkylating agent [31], and gamma radiation that causes the breaking of double-stranded DNA [32] was evaluated. We found a relationship between Bz sensitivity and sensitivity to these genotoxic agents, similar to the previous report of Rajao *et al*. [3]. Additionally, it was found that the transfected parasites treated with Bz showed the retention of the cell cycle in the G1 phase, possibly as a result of the genotoxic and cytostatic effect previously reported for Bz [4,5].

**Figure 6.**
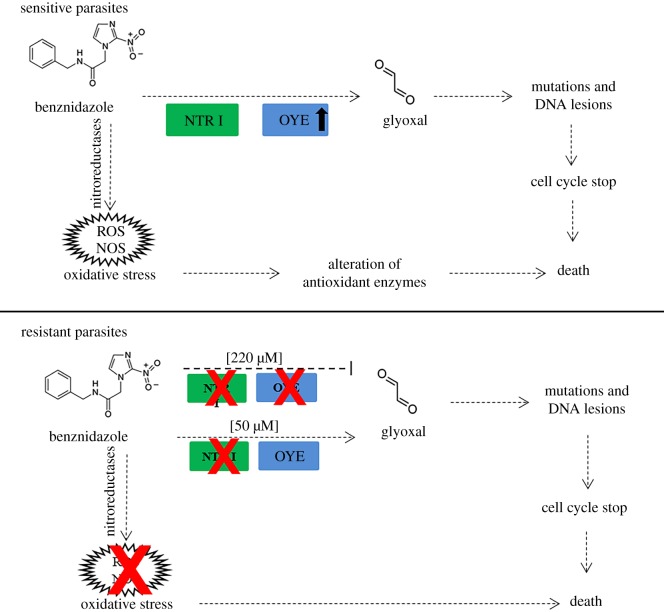
Proposed model for OYE participation in the mechanism of action of Bz. Bz-sensitive *T. cruzi* parasites have functional NTR I and OYE, which would participate in the activation of Bz and in the generation of toxic intermediates, such as glyoxal and reactive species, that would cause genetic damage and oxidative stress. The overexpression of OYE in these parasites increases sensitivity to Bz, H_2_O_2_ and genotoxic agents, which leads to death. In parasites resistant to Bz 50 µM (lacking NTR I), OYE enzyme is overexpressed, reducing the production of toxic intermediates to the parasite. These resistant parasites did not survive OYE overexpression. OYE is also not functional at higher concentrations of Bz (220 µM) as was reported by Murta *et al*. [14].

The results found in this work with OYE suggest that this enzyme is involved in the activation pathway of Bz because its overexpression in sensitive parasites increased the sensitivity to Bz, as well as to the oxidative stress and genetic damage. We propose that OYE is participating, together with NTR I, in the Bz activation pathway. Thus, when susceptible parasites are stimulated to overexpress this protein they become more sensitive to Bz and oxidative stress (figure 6). Conversely, parasites with induced resistance to Bz, which have no NTR I active, already show OYE overexpression. The additional overexpression produces non-viable parasites. Thus, it supports previously reported results by other authors who have linked this gene with the mechanism of action of Bz, specifically with the activation and production of toxic metabolites [12–14]. Future studies could be conducted to evaluate the gene expression profiles associated with DNA damage repair by comparing parasites sensitive and resistant to Bz and the relationship to sensitivity to oxidative stress.

## Supplementary Material

Figure S1

## Data accessibility

All data for this study are found in this paper and in the electronic supplementary material.
