# Supplementary figures and images for: Prostaglandin F2α synthase in Trypanosoma cruzi plays critical roles in oxidative stress and susceptibility to benznidazole
Source: R Soc Open Sci. 2017 Sep 20;4(9):170773. doi: 10.1098/rsos.170773 (PMC5627119; doi:10.1098/rsos.170773)

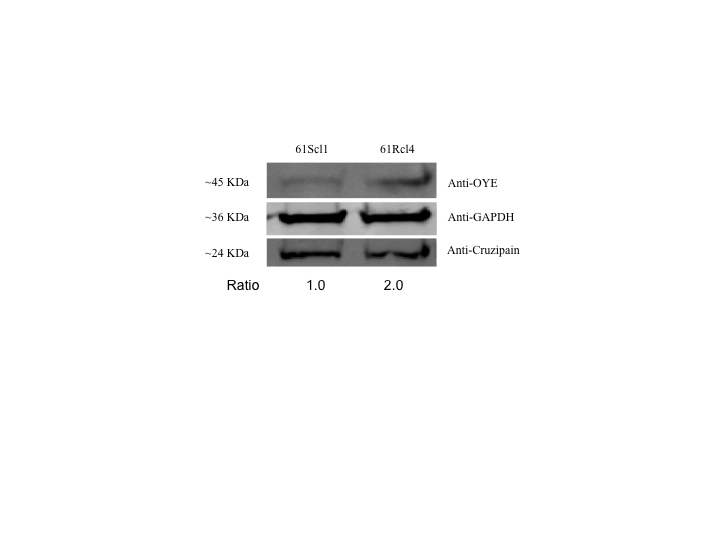

Supplement: Figure S1 [file rsos170773supp1.tif]
